# Using Nuclear Genomic Data to Address Intractable Relationships and Gene Tree Discordance in an Ancient Group of Gymnosperms (*Ephedra*, Gnetales)

**DOI:** 10.1002/ece3.73863

**Published:** 2026-06-30

**Authors:** Ruben Blokzijl, Catarina Rydin

**Affiliations:** ^1^ Department of Ecology, Environment and Plant Sciences Stockholm University Stockholm Sweden

**Keywords:** nuclear DNA, paralogy, phylogenetic discordance, phylogenomics, recombination, target capture

## Abstract

The evolutionary history of *Ephedra* L. has over time proved to be a very difficult phylogenetic problem, something which probably is best reflected by the incongruent phylogenetic relationships demonstrated in and among previous studies. A low number of phylogenetically informative sites and different rooting strategies have been suggested as explanations, but the role of gene tree discordance and its underlying causes and influence on phylogenetic reconstruction in *Ephedra* have largely remained unexplored. In the present study, we address the phylogeny of *Ephedra* using information from a large set of nuclear low‐copy genes obtained with a specifically designed bait set. We explore the potential effect of putative paralogy, intra‐locus recombination, and chimeric sequences on phylogenetic estimation in *Ephedra*, while striving to minimize the risk for gene tree error. Our results indicate that recombination and putatively paralogous sequences have limited influence on phylogenetic results, although handling of putative paralogs and recombinants did improve overall species tree resolution and statistics. A robust *Ephedra* species phylogeny was obtained by analyzing gene trees, in which orthologous clades were inferred from data composed of orthologous and putatively paralogous sequences. The results support the division of *Ephedra* into three geographically defined clades, with the American clade as sister to the Mediterranean clade and the Asian clade. Relationships among species within these three clades were relatively consistent among our analytical approaches, but our results highlighted moderate to strong signals of gene tree discordance at many branches including the deepest split. Incomplete lineage sorting is a possible explanation for this discord for many branches, but hybridization/introgression is also commonly indicated.

## Graphical abstract

Low‐copy nuclear genes were used to address the notoriously difficult phylogeny of *Ephedra*. Additionally, we explored the potential effect of putative paralogy, intra‐locus recombination, and chimeric sequences on phylogenetic estimation.

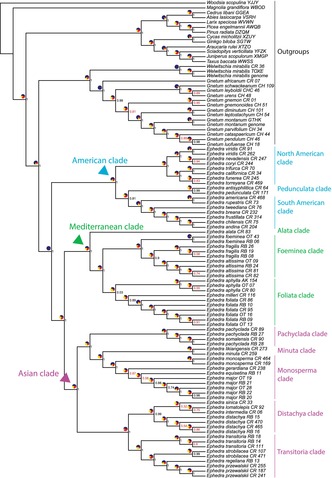

## 1 Introduction

**FIGURE 1:**
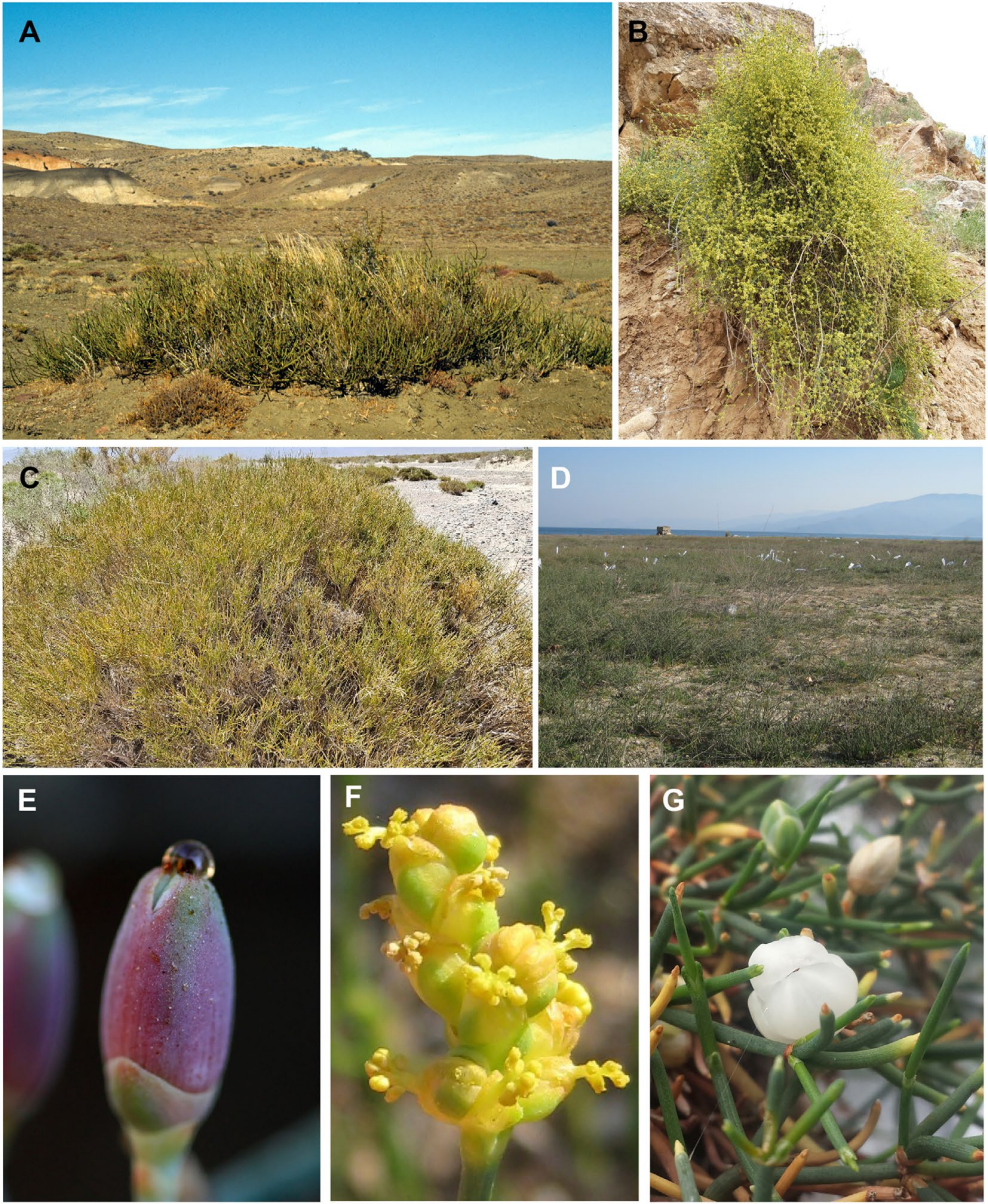
*Ephedra* (Gnetales). (A) *Ephedra* shrub, Patagonia (Photo: David Cantrill). (B) *Ephedra aphylla*, male plant hanging from cliff, Jordan (Photo: Ruben Blokzijl). (C) *Ephedra* shrub, Qinghai Province, China (Photo: Qin Yuan). (D) *Ephedra distachya*, soil‐ binding creeping habit, Greece (Photo: Kristina Bolinder). (E) *Ephedra foeminea*, female cone with pollination drop (Photo: Kristina Bolinder). (F) *Ephedra distachya*, male cone (Photo: Kristina Bolinder). (G) *Ephedra frustillata*, mature female cone (Photo: Catarina Rydin).

*Ephedra* L. (Figure 1) comprises the gymnospermous order Gnetales together with its sister genera *Welwitschia* Hook.f. and *Gnetum* L. Upon first sight these three genera look very dissimilar in gross appearance and they have been argued to be remnants of a former much greater diversity that probably peaked during the Early‐mid Cretaceous (e.g., Crane 1988; Rydin et al. 2004; Friis et al. 2011; Barbolini et al. 2025). Despite the differences, they do share morphological characters like strictly opposite and decussate or whorled branching and leaf arrangement, a compound cone structure, a micropylar tube formed by the integument, and extra‐ovular structures (Endress 1996; Rydin et al. 2010). Molecularly they are undisputed as an order; they are always resolved as a monophyletic group, although their relationship to other seed plants has remained elusive (Smith and Brown 2018).

There are about 50–60 extant species of *Ephedra*, and they inhabit warm arid regions around the world except in southern Africa and Australia (Ickert‐Bond and Renner 2016). Overall growth habit ranges from tiny individuals to large upright or scrambling shrubs, even small trees, with reduced leaves and functionally unisexual compound cones found on male and female individuals, respectively. In contrast to their relatively well‐documented morphology and anatomy (e.g., Thoday and Berridge 1912; Pant and Mehra 1964; El‐Ghazaly et al. 1998; Rydin et al. 2010; Bolinder, Norbäck Ivarsson, et al. 2016; Barbolini et al. 2025), general genetic features of *Ephedra* are still poorly known. Flow cytometry analyses have highlighted that the genome sizes in *Ephedra* are very large, with *E. antisyphilitica* having the largest estimated genome size (37,5Gb/1C) across *Ephedra* species and perhaps even across gymnosperms (Ickert‐Bond et al. 2020), and a fully annotated reference genome has still not been produced. Nevertheless, significant progress has been made over the last decades to understand the evolutionary drivers that have shaped the current and former diversity of *Ephedra* (Ickert‐Bond and Wojciechowski 2004; Rydin et al. 2004, 2010, 2021; Huang et al. 2005; Ickert‐Bond et al. 2009, 2020; Rydin and Korall 2009; Kakiuchi et al. 2011; Loera et al. 2012, 2015; Qin et al. 2013; Bolinder et al. 2015; Bolinder, Humphreys, et al. 2016; Bolinder, Norbäck Ivarsson, et al. 2016; Ickert‐Bond and Renner 2016; Wu et al. 2016, 2021; Yu et al. 2023; Barbolini et al. 2025). In addition to studies of phylogeny, divergence times of clades and evolution of features, polyploidy has been shown to prevail in *Ephedra*, with approximately 83% of the species being polyploid or having polyploid cytotypes (Ickert‐Bond et al. 2020). Based on two chloroplast markers and two nuclear markers, Wu et al. (2016) suggested that allopolyploidy was a dominant mode of speciation in *Ephedra* and this finding was later supported by transcriptomic information from two Asian *Ephedra* species (Wu et al. 2021).

However, notwithstanding ample previous efforts, the phylogeny of *Ephedra* and its relationship to its Mesozoic ancestors have remained unclear, and has proven to be a very difficult phylogenetic problem (e.g., Rydin et al. 2021). Ample questions remain unanswered, including the deepest split in the genus. Most phylogenetic analyses of *Ephedra* have been based on data from the nuclear ribosomal region and/or plastid information. The plastome can generally be considered a single independently evolving locus (Doyle 2022), and the same is basically true for the nuclear ribosomal DNA due to the homogenizing process of concerted evolution (Elder and Turner 1995). Effectively, this means that estimations of the *Ephedra* phylogeny have mostly been based on two genes or gene trees; only in a few studies have low copy nuclear genes been used (Wu et al. 2016; Yu et al. 2023). Accordingly, the potential impact of evolutionary drivers like gene duplication and loss, incomplete lineage sorting (ILS) and recombination on the ephedran phylogeny has not been sufficiently studied. Furthermore, *Ephedra* constitutes an excellently suited study system for general investigations of gene tree heterogeneity due to a number of biological and non‐biological sources, because of its low sequence divergence among species shown in many previous studies (e.g., Ickert‐Bond and Wojciechowski 2004; Rydin et al. 2004; Huang et al. 2005; Rydin and Korall 2009), a potentially rapid speciation in the living clade (Huang and Price 2003; Rydin et al. 2021), and the frequent occurrence of polyploidy that makes *Ephedra* unique among gymnosperms since polyploidy is otherwise only naturally occurring in a few gymnospermous genera (Wan et al. 2022).

Data production using target enrichment, that is, isolation of preselected DNA regions from larger genomic regions, has become a go‐to strategy to retrieve large amounts of genomic information to address phylogenetic questions at different hierarchical and evolutionary scales (see e.g., Ogutcen et al. 2024; Yan et al. 2024). The method has the advantage that subsequent sequencing efforts are focused on pertinent DNA sequences, and depending on the strategy chosen, for example, in solution hybrid capture (Gnirke et al. 2009), it can even become easily scalable and cost efficient to employ (Grover et al. 2012; Jones and Good 2016; McKain et al. 2018). The field of molecular systematics has thus also evolved from the traditional use of data from a few loci often analyzed with a concatenation approach to investigations of evolutionary histories of organisms, to utilization of massively larger datasets comprising hundreds to thousands of nuclear loci, entire organellar genomes and/or the nuclear ribosomal cistron. With the accessibility to large quantities of genomic information, the possibility to analyze individual gene trees and investigate discord between them have improved dramatically.

Systematic biology has largely adopted the multispecies coalescent model (MSCM; Pamilo and Nei 1988; Degnan and Rosenberg 2009) and coalescent‐based methods (generally classified into summary methods, co‐estimation methods, and site‐based methods (Mirarab et al. 2021)) as common practice for species tree estimation and investigations of topological discordance. Incomplete lineage sorting of genetic polymorphism in ancestral species is a common reason for gene tree discordance (Pamilo and Nei 1988; Mirarab et al. 2014) since it leads to temporal mismatch between gene divergence and species divergence, resulting in gene trees that differ from each other and from the evolutionary history of the species (Maddison 1997). As implemented in analytical tools for species tree inference (Molloy and Tandy 2023), the MSC models ILS and allows each locus to have its own evolutionary history within the boundaries of the species.

Besides ILS, gene duplication and loss, and horizontal gene transfer (including introgression and hybridization) are common in plants and considered important biological sources for gene tree discordance (Rieseberg and Soltis 1991; Maddison 1997). Several processes may lead to gene duplication that produces paralogs—gene copies that within the concept of homology have been able to evolve and descend independently over time (Fitch 1970; Koonin 2005)—with polyploidy as one of the identified mechanisms that introduces paralogs to the genome via whole genome duplication (Sémon and Wolfe 2007). Paralogous sequences may mislead phylogenetic reconstruction because they do not derive from speciation events like orthologs (Fitch 1970; Yang and Smith 2014), but they can be useful if handled correctly as they may contain information on evolution events that happened after the duplication event (Hellmuth et al. 2015; Smith and Hahn 2021).

Further, recombination is a source of variation in sexually reproducing organisms that can cause reticulate patterns within genes and induce inaccurate phylogenetic estimates (Lanier and Knowles 2012; Wang and Liu 2016). While the MSCM assumes independent evolution between loci, recombination within loci violates the assumptions of both concatenation methods and coalescent‐based methods such as ASTRAL (Lanier and Knowles 2012; Mirarab and Warnow 2015; Doyle 2022). Ignoring intra‐locus recombination can thus cause error in species tree estimation (Lanier and Knowles 2012), although it has also been argued that gene tree discordance due to intra‐locus recombination is negligible in practice (Edwards et al. 2016).

In addition to biological processes, non‐biological sources of discordance can introduce error in gene tree estimations (Roch and Warnow 2015), caused for example by factors like incorrect or insufficient phylogenetic information and few or short sequences (loci). While difficult to unambiguously demonstrate in studies based on real data, the risk for gene tree estimation error can thus be mitigated for example by adding information in addition to deleting uncertain or fragmentary parts of the alignment and collapsing poorly supported branches before species tree estimation using summary methods (Sayyari et al. 2017; Zhang et al. 2018).

Species tree estimation using the MSCM contrasts with the concatenation approach, which assumes that all loci have the same evolutionary history and evolved under the same species topology (Degnan and Rosenberg 2009). Studies have shown that using a concatenation approach, for example by analyzing a large alignment that comprises many loci based on maximum likelihood, is not statistically consistent under the MSCM and can be positively misleading (Roch and Steel 2015). The choice between coalescent‐based methods and a concatenation approach to address phylogenetic questions has, however, not been without controversy (Gatesy and Springer 2014; Mirarab et al. 2014). A concatenation approach can be preferable if gene tree discordance is low or the risk for gene tree error is high (Mirarab and Warnow 2015). Less has also been known about suitable analytical approaches when sources other than ILS (or multiple sources) of gene tree discordance exist but it has been argued that the coalescent‐based summary method ASTRAL (Mirarab et al. 2014; Zhang et al. 2018) has some robustness to gene tree discordance of several kinds (Lanier and Knowles 2012; Davidson et al. 2015; Legried et al. 2021).

With the aim to address the longstanding questions on evolutionary relationships in *Ephedra*, we sampled nearly a thousand putatively independently evolving low‐copy nuclear exon loci representing several hundred genes produced using target capture and a new bait set specifically designed for *Ephedra*. We investigated levels of gene tree discordance and the presence and potential impact of ILS, recombination, and paralogous sequences on the robustness of the phylogenetic reconstruction using the multispecies coalescent model as implemented in ASTRAL‐III as well as a concatenation method. Since paralogous and recombinant sequences as well as ILS have the potential to seriously influence phylogenetic inference, understanding the extent of their presence and contribution to gene tree discordance in *Ephedra* may explain the disparate results in previous phylogenetic work and reveal some of the drivers that shaped evolution in this ancient lineage.

## 2 Materials and Methods

### 2.1 Taxon Sampling

We selected 68 *Ephedra* specimens for target enrichment, representing 39 species and the global diversity of the genus. In addition, 12 samples of *Gnetum* and one sample of *Welwitschia* were selected to represent the remaining Gnetales. The specimens were sampled from living material as well as from the collections in the herbaria BM, BR, CAI, E, L, MO, NY, O, S, UC, UPS, W, WU, and Z. In addition, transcriptomic data from the 1KP project (Carpenter et al. 2019) were retrieved for 14 seed plant specimens (one representing *Gnetum*, one *Welwitschia*, five pinaceous conifers, four cupressophyte conifers, and one each representing cycads, *Ginkgo*, and angiosperms), and genomic data were downloaded for one specimen of *Gnetum montanum* and *Welwitschia mirabilis* (Liu et al. 2021). The polypod fern *Woodsia* (transcriptomic data from the 1KP project) was used as the ultimate outgroup. Furthermore, transcriptomic data of *E. equisetina* and *E. sinica* (1KP project), as well as genome skimming data of *E. foeminea* RB 06, were selected and used for bait design. A full overview of all sequence data used, including our sample of 98 terminals and corresponding voucher and accession number information, is given in Table S1.

### 2.2 Bait Design

Single copy genes from the universal angiosperm 353 bait kit (Johnson et al. 2019) and the seed plant study by Li et al. (2017) were selected as basis upon which the *Ephedra* baits were designed. Transcriptomic data of *E. equisetina* and *E. sinica* were mapped against the reference sequences as translated amino acids in six frames using BLAT v36 (Kent 2002) to find corresponding gene sequences in *Ephedra*. Sequence depth was obtained by mapping genome skimming data of *E. foeminea* RB 06 to the *Ephedra* transcripts with Bowtie2 v2.3.5.1 (Langmead and Salzberg 2012) using local settings. Mapping performance and inference of suitable DNA regions for bait design was checked in Gap5 from the Staden Package v2.0.0 (Bonfield and Whitwham 2010), as was the extraction of the suitable DNA regions. Extracted DNA sequences were reverse complemented with seqtk v1.3‐r106 (Li 2018), if needed, and checked for similarity and repetitiveness with cd‐hit‐est v4.8.1 (Li and Godzik 2006) using a 90% cutoff and RepeatMasker v4.1.0 (Smit et al. 2019), respectively. The final DNA sequences, now target sequences, were sent to Daicel Arbor Biosciences, Ann Arbor, MI, United States for synthesis of our bait set, the Ephedra241 bait set, with 80 bp biotinylated RNA probes and a 3× tiling.

### 2.3 DNA Extraction, Library Preparation and Target Enrichment

Young *Ephedra* shoots were sampled from living‐ and herbarium material. The samples were pulverized with stainless steel beads using a TissueLyser LT (Qiagen, Hilden, Germany) and DNA was extracted with a cetyltrimethylammonium bromide (CTAB) extraction method (Doyle and Doyle 1987). After assessing fragment size distribution on agarose gel (1%) electrophoresis, DNA samples were sheared to a set target insert size of 350 bp with a Covaris E220 Focused‐ultrasonicator (Covaris, Woburn, MA, United States) at Science for Life Laboratory (Solna, Sweden).

DNA libraries were prepared with a TruSeq Nano DNA library preparation kit (Illumina, San Diego, CA, United States) according to the manufacturer's protocol (v June 2015), but with minor modifications: the order of the size selection and the end repair steps were switched to reduce the loss of resources during the library preparation process, and for most cleaning steps a ratio of 0.8:1 of SPRI beads (Beckman Coulter, Brea, CA, United States) to reaction volume was used. Libraries were quantified with the Qubit dsDNA High Sensitivity (HS) Assay Kit on a Qubit 2.0 Fluorometer (Thermo Fisher Scientific, Waltham, MA, United States), and fragment size distributions were assessed with High Sensitivity DNA chips and reagents on a 2100 Bioanalyzer (Agilent Technologies, Santa Clara, CA, United States).

Guided by library concentration and average fragment size, libraries were multiplexed into pools of 5‐, 6‐ or 8‐plexes. Each pool contained approximately 500 ng of library and was concentrated with a miVac (Genevac, Ipswich, United Kingdom) at approximately 40°C prior enrichment. Pools, several *Ephedra* pools and two outgroup pools, were enriched with the biotinylated RNA probes from the Ephedra241 bait set according to the manufacturer's protocol v4 (Daicel Arbor Biosciences, Ann Arbor, MI, United States) and let to hybridize for 16–18 h at 65°C (*Ephedra* pools) or 62°C (outgroup pools). The enriched library products were amplified with KAPA HiFi (2×) HotStart ReadyMix PCR Kit (Roche, Basel, Switzerland) for 18 cycles and subsequently cleaned with a ratio of 0.8:1 of AMPure XP beads (Beckman Coulter, Brea, CA, United States) to reaction volume. Quantification and fragment size distribution of the enriched libraries were measured as indicated above.

### 2.4 Sequencing and Sequence Data Pre‐Processing

Enriched library pools were normalized and multiplexed at equimolar concentrations prior sequencing. Samples were sequenced with a 2 × 151 bp setup on a NextSeq500 using ‘Mid‐Output’ chemistry, or on a NovaSeq6000 using ‘NovaSeqXp’ workflow in ‘SP’ mode (Illumina, San Diego, CA, United States) at Science for Life Laboratory (Solna, Sweden). Subsequent demultiplexing of the data and Bcl to FASTQ conversion using bcl2fastq_v2.20.0.422 from the CASAVA software suite was also performed at Science for Life Laboratory. Raw read files were preprocessed with the clumpify.sh and bbduk.sh scripts from the BBTools suite v38.69 (Bushnell 2014). Clumpify.sh was used to remove duplicated reads from the data files, while bbduk.sh was used to trim reads for leftover adapter sequences and low‐quality bases (< Q15). Reads shorter than 36 bp were removed from the data files. See Table S2 for statistics on sequencing and data filtering.

### 2.5 Data Assembly

A common feature in gymnosperms are long intron sequences between exon sequences that make up a gene (Wan et al. 2022). Failing to capture these long intron sequences increases the probability for stitched (i.e., artificially assembled, chimeric) gene sequences. Therefore, given our short‐read data and initial gene sequence observations, we consider all obtained gene sequences in *Ephedra* to potentially be of stitched nature. To account for this and its potential influence on downstream phylogenetic inference, we assembled the following two types of data: gene supercontig sequences and exon supercontig sequences, with a supercontig sequence being a sequence that contains non‐coding flanking regions (introns, ′5 and ′3 ends of genes) besides their protein coding region(s). We treat both a gene supercontig sequence and an exon supercontig sequence as a locus that has evolved independently.

Assembly of the gene‐ and exon coding sequences was performed with the HybPiper assembly pipeline v1.3 (Johnson et al. 2016), which for each target sequence aligns it with the preprocessed sequence data using BWA v0.7.17‐r1188 (Li and Durbin 2009), collects the reads into their respective directory, then assembles the reads into contigs with SPAdes v3.13.1 (Bankevich et al. 2012), and aligns the contigs back to their target sequence to infer exon‐intron boundaries using exonerate v2.4.0 (Slater and Birney 2005). Post assembly script paralog_investigator.py was run to infer the presence of putatively paralogous sequences, while the intronerate.py script was run to generate the intron and supercontig sequences. In addition, assembly statistics were retrieved with the post‐assembly scripts get_seq_lengths.py and hybpiper_stats.py, and can be found in Table S3.

Generating exon supercontig sequences resulted in cases where neighboring loci shared the same small stretch of intron information. To identify these loci an all‐against‐all BLAST search (Altschul et al. 1990) was conducted. Coordinates of the BLAST mapping were used to trim the sequences that contained the same stretch of intron accordingly, thereby keeping the exon's coding region of the trimmed sequences intact. This was performed for species *E. foeminea* OT 43, which trimmed exon supercontig sequences then were used as reference for another assembly round with HybPiper (as described above) with the remaining accessions.

For the assembly of target captured *Gnetum* and *Welwitschia* samples, translated target sequences of *Ephedra* were used as reference sequences, while BLASTX v2.9.0+ (Camacho et al. 2009) was used to map the preprocessed sequence data against these reference sequences. Other steps in the HybPiper assembly pipeline remained the same. Additionally, transcriptomic data from the 1KP project and genomic data of *Gnetum montanum* and *Welwitschia mirabilis* were searched to complement the target captured outgroup sequences. For each taxon, its transcriptomic or genomic data was mapped against the *Ephedra* gene‐ and exon coding sequences as translated amino acids in six frames using BLAT. The corresponding region was extracted from the matching transcript or contig using extractseq as part of the EMBOSS suit v6.6.0.0 (Rice et al. 2000) based on the BLAT mapping coordinates and reverse complemented (if needed) with seqtk. Only loci that have at least one outgroup sequence (coding sequences only) have been considered for phylogenetic analyses.

### 2.6 Dataset Cleaning and Alignment

A total of six datasets were created (Tables 1 and 2). The datasets specifics and their purpose(s) will be described below in their own respective paragraphs. Irrespectively, for each dataset, alignment of the *Ephedra* sequences was performed with MAFFT v7.525 using the ‐‐auto argument, which automatically selects the best fitting alignment strategy (Katoh and Standley 2013). To improve data quality and reduce the risk for misalignment and gene tree error, the datasets were trimmed by removing accession‐specific stretches of sequences that were erroneous as identified with TAPER v1.0.2 (Zhang et al. 2021), by removing columns with < 20% site occupation with phyx (Brown et al. 2017), and finally, by removing accessions with a sequence length less than one‐third of the total alignment length. Loci with a taxon coverage of less than 30 unique taxa were excluded from downstream usage. After another round of alignment, gene trees were reconstructed (see below). We define a gene tree as a phylogeny estimation based on data from a single locus irrespective of its content. Outlier sequences were identified and trimmed from the gene trees and respective alignments with TreeShrink (Mai and Mirarab 2018), using a false positive error of α = 0.05 under the per‐gene mode. After cleaning up the *Ephedra* alignments, outgroup sequences were added, followed by another round of aligning and column trimming (no outlier detection), as described above. Alignment statistics were obtained with the summary command using AMAS (Borowiec 2016) (see Table 1 for selected alignment statistics and Table S4 for the full).

**TABLE 1:** Selected statistics of the datasets used for phylogenetic inference in this study.

	Dataset	Characteristic(s)	Species tree estimation analysis	No. loci	Average locus length	Average no. PIS per locus	Total no. sites	Total no. PIS (%)	Average taxon occupancy
						Ingroup|Outgroup		Ingroup|Outgroup	Ingroup|Outgroup
1	*Paralog ortholog‐inferred* ^a^ (Figure 2)	Loci and gene trees include orthologous and paralogous sequences, orthologous clades inferred from gene trees	ASTRAL‐III	882	482	50|109	424,791	44,513 (10%)|95,756 (23%)	49|65
1	*Paralog ortholog‐inferred* ^a^ (Figure 3)	Loci and gene trees include orthologous and paralogous sequences, orthologous clades inferred from gene trees	IQ tree (ML concatenation)	882	482	50|109	424,791	44,513 (10%)|95,756 (23%)	49|65
2	*Paralog 1to1 ortholog* ^a^ (Figure S1)	Loci with paralog warnings removed entirely	ASTRAL‐III	114	587	75|157	66,974	8604 (13%)|17,861 (27%)	66|83
3	*Paralog AstralPro* ^a^ (Figure S2)	Loci and gene trees include orthologous and paralogous sequences, multiple sequences per accession	ASTRAL‐Pro	978	472	81|134	461,845	79,516 (17%)|131,035 (28%)	80^b^|96^b^
4	*Exon supercontig standard* (Figure S3)	Exons + regions flanking the exons, single sequence per accession (standard output from HybPiper)	ASTRAL‐III	979	474	66|121	464,012	64,871 (14%)|118,065 (25%)	67|82
5	*Exon supercontig recombination‐free* (Figure S4)	Loci with evidence for recombination removed entirely	ASTRAL‐III	249	348	48|87	86,720	11,880 (14%)|21,773 (25%)	67|81
6	*Gene supercontig* (Figure S5)	Exons, introns + regions flanking the gene	ASTRAL‐III	240	2812	560|871	674,994	134,486 (20%)|209,137 (31%)	65|88

Abbreviation: PIS, parsimony informative sites.

^a^ The paralog datasets are generated from exon supercontig data.

^b^ Multiple sequences per accession are possible.

**TABLE 2:** Selected statistics of the gene trees and species trees inferred in this study.

	Dataset	Characteristic(s)	Species tree estimation analysis	No. loci	Normalized Quartet Score (NQS)	Average branch support (LPP)	No. branches with ≥ 95% support	No. branches for which a polytomy could not be rejected^b^	Average UFBoot in input gene trees
						Ingroup|Outgroup	Ingroup|Outgroup	Ingroup|Outgroup	Ingroup|Outgroup
1	*Paralog ortholog‐inferred* ^a^ (Figure 2)	Loci and gene trees include orthologous and paralogous sequences, orthologous clades inferred from gene trees	ASTRAL‐III	882	0.71	0.91|0.94	46|72	6|10	71|72
1	*Paralog ortholog‐inferred* ^a^ (Figure 3)	Loci and gene trees include orthologous and paralogous sequences, orthologous clades inferred from gene trees	IQ tree (ML concatenation)	882	n/a	0.95^c^|0.93^c^	61|84	n/a	n/a
2	*Paralog 1to1 ortholog* ^a^ (Figure S1)	Loci with paralog warnings removed entirely	ASTRAL‐III	114	0.69	0.81|0.83	27|45	22|30	71|73
3	*Paralog AstralPro* ^a^ (Figure S2)	Loci and gene trees include orthologous and paralogous sequences, multiple sequences per accession	ASTRAL‐Pro	978	n/a	0.91|0.92	48|71	n/a	64|66
4	*Exon supercontig standard* (Figure S3)	Exons + regions flanking the exons, single sequence per accession (standard output from HybPiper)	ASTRAL‐III	979	0.68	0.95|0.95	51|76	7|12	71|72
5	*Exon supercontig recombination‐free* (Figure S4)	Loci with evidence for recombination removed entirely	ASTRAL‐III	249	0.67	0.88|0.86	39|57	15|25	70|71
6	*Gene supercontig* (Figure S5)	Exons, introns + regions flanking the gene	ASTRAL‐III	240	0.72	0.89|0.90	43|67	13|18	79|81

Abbreviations: LPP, local posterior probability; UFBoot, ultrafast bootstrap approximation.

^a^ The paralog datasets are generated from exon supercontig data.

^b^ Only interspecific polytomies are considered.

^c^ Bootstrap support values.

### 2.7 Gene Tree Reconstruction and Phylogenetic Analyses

To be able to investigate gene tree discordance and reasons thereof, we mainly employed species tree estimation using the multispecies coalescent model as implemented in the quartet‐based summary method ASTRAL‐III v5.7.8 (Zhang et al. 2018). Gene trees were inferred with IQ‐TREE v3.0.1 (Wong et al. 2026) under the sequence evolution model selected by ModelFinder (Kalyaanamoorthy et al. 2017) and assessed using 1000 ultrafast bootstrap replicates (Hoang et al. 2018) as both are implemented in IQ‐TREE. Branches of gene trees with less than 20% bootstrap support were collapsed with Newick utilities v1.6 (Junier and Zdobnov 2010). The final gene trees were summarized into a species tree in ASTRAL‐III. Branch support was assessed with local posterior probability (LPP; Sayyari and Mirarab 2016). The quartet frequencies of alternative topologies were used for the assessment of tree discordance, which were obtained with the ‐t2 argument in ASTRAL‐III. A polytomy test, a statistical test to test if the null hypothesis that a given branch should be considered a polytomy can be rejected, was conducted with the ‐t10 argument in ASTRAL‐III. The normalized quartet score (NQS) is provided for each analysis. It quantifies gene tree discordance within a species tree by counting the number of quartet trees within the gene trees that are also present within the species tree divided by the total number of quartet trees within the gene trees (Mirarab 2023). Notable differences from the analytical generalization described above will be stated in the dataset specific paragraphs below.

All species trees were rooted on the polypod fern *Woodsia scopulina* . The final species trees were plotted using R (R Core Team 2024) packages treeio v1.30 (Wang et al. 2020) and ape v5.8–1 (Paradis and Schliep 2019), while package TreeDist v2.9.2 (Smith 2020b) was used to calculate the Normalized Robinson‐Foulds (NRF) (Smith 2020a). The NRF provides a normalized score of the Robinson‐Foulds distance, which quantifies the difference between two phylogenetic trees by counting the differing bipartitions providing identical taxon sets in both trees (Robinson and Foulds 1981).

### 2.8 The Ephedra Phylogeny—Dataset 1

Multiple paralog warnings were issued by HybPiper for each accession during the assembly of the exon supercontig sequences, which combined with the polyploidy status of many species makes it likely that many assembled sequences are putatively paralogous sequences. A paralog warning is issued when multiple sequences are assembled that span 85% of the reference sequence length (Johnson et al. 2016). Consequently, we treated those sequences as such and adopted the ortholog inference workflow and scripts from Morales‐Briones et al. (2022) to utilize the putatively paralogous sequences as part of the phylogenetic inference process. A paralog‐based dataset was constructed using all the assembled exon supercontig sequences (orthologous and putatively paralogous) of *Ephedra* accessions, which were retrieved with the HybPiper post‐assembly script paralog_retriever.py, followed by the alignment and dataset cleaning steps.

Gene trees were reconstructed from alignments containing both orthologous and putatively paralogous sequences, and orthologous clades were inferred and pruned from these gene trees using the rooted tree (RT) approach when at least 30 unique ingroup taxa are present. With the RT approach, gene trees are rooted on outgroups, iteratively searched for subtrees that meet the taxon occupancy criteria, and pruned. A taxon occupancy of approximately 50% per locus is used to reduce the negative effects of partial taxon coverage on phylogenetic inference (Steenwyk et al. 2023), while keeping orthologous gene trees. Outgroup sequences that were trimmed during the pruning of orthologous clades were added again, creating the *paralog ortholog‐inferred* dataset (dataset 1).

In addition to ASTRAL‐III, a maximum likelihood (ML) estimation was applied to analyze this dataset. Maximum likelihood analysis can be preferred over coalescent analysis when gene tree estimation errors are suspected, a scenario that is possible in this case given the reduction in taxon occupancy in the dataset in combination with the general information poverty in molecular datasets of *Ephedra* shown in previous studies. The concatenated version of the dataset was produced using AMAS with the concat command. The ML analysis was run with IQ‐TREE, with the data partitioned by loci and with each partitioning having its own evolutionary model selected by ModelFinder. Branch support was assessed using 1000 ultrafast bootstrap replicates.

### 2.9 Additional Treatment of Putative Paralogs—Datasets 2–4

To assess the influence of putatively paralogous sequences on the robustness of species tree estimation we employed three additional paralog‐handling strategies and compared the species tree topologies and statistics of these strategies with each other. Two of these datasets and strategies were also produced from the paralog‐base dataset described above (datasets 2–3). Gene supercontig sequences were not considered for assessment of paralogy as the number of putatively paralog sequences generated is likely biased given the stitched nature of the sequences.

For the *paralog 1to1 ortholog* dataset (dataset 2), all loci with indications of putatively paralogous sequences (loci with paralog warnings generated by HybPiper) were left out from species tree estimation. The *paralog AstralPro* dataset (dataset 3) was directly obtained from the paralog base dataset and served as input for the paralog‐tolerant coalescent method ASTRAL‐Pro v1.15.1.3 (Zhang and Mirarab 2022). The argument ‐u2 was used to obtain the quartet frequencies of alternative topologies.

The results of these paralog handling strategies were compared with those of the *exon supercontig standard* dataset (dataset 4), which is composed of the standard single exon sequence output from HybPiper. This dataset can be considered a paralog control dataset, wherein putatively paralogous sequences are a priori considered to have a negligible effect on phylogenetic inference in *Ephedra*.

### 2.10 Treatment of Recombinant Sequences—Datasets 5–6

Gene supercontig sequences of artificially recombinant nature would, if present, influence downstream analysis, and therefore we applied two strategies to assess if artificial and/or biological intra‐locus recombination affects species tree estimation in *Ephedra*. First, a *gene supercontig* dataset (dataset 6) was generated composed of gene supercontig sequences from HybPiper. These gene supercontig sequences were split into smaller units (exon supercontig sequences) to overcome the effects of recombination; the *exon supercontig standard* dataset (dataset 4, described above).

For the second strategy, a Phi Test (Bruen et al. 2006) as implemented in SplitsTree v6.4.11 (Huson and Bryant 2024) was employed to quantify the extent of recombination in our *exon supercontig standard* dataset (dataset 4) and *gene supercontig* dataset (dataset 6), using only the *Ephedra* alignments (i.e., no outgroups included). Loci that did not show evidence of recombination, meaning a statistical *p* ≥ 0.05 (no significance) in the Phi Test, were used to generate a recombination‐free dataset with outgroups. However, only the *exon supercontig recombination‐free* dataset (dataset 5) was used for downstream analysis since only a few loci in the gene supercontig dataset were estimated to be recombination‐free. The topologies and statistics of all respective datasets were compared.

For simplicity and considering the relatively similar topologies resulting from the two analytical strategies employed for dataset 1, we chose to exclusively analyze the additional datasets for further assessment of paralogy and recombination (datasets 2–6) using species tree estimation in ASTRAL.

## 3 Results

### 3.1 Bait Set and Sequencing Statistics

The Ephedra241 bait set successfully targeted 1052 exons comprising 241 genes. On average, 17% of the reads in the filtered datafiles of *Ephedra* samples mapped to the target exon sequences and provided an exon coverage of 691×. For the target enriched outgroup samples of *Welwitschia* and *Gnetum*, an average exon coverage of 21× was obtained from < 1% of the filtered reads that were on target. Full statistics on sequencing and data filtering are given in Table S2.

### 3.2 Assembly Statistics

Across all *Ephedra* samples, on average, 1009 exon coding sequences were assembled that constituted at least 75% of the target sequence length, whereas for *Gnetum* and *Welwitschia* samples, on average, 205 exons had assembled coding sequences for at least 75% of the target sequence length. For gene coding sequences, on average, 175 and 97 sequences were assembled that constituted at least 75% of the target sequence length for *Ephedra* and outgroup samples, respectively. Paralog warnings were generated for most accessions during the assembly of gene coding sequences (an average of 14 per accession) and for all accessions during the assembly of exon coding sequences (an average of 329 per accession). In the case of *Ephedra*, the number of paralog warnings varied among species, which is most evident in exon coding sequence assemblies. For example, the six *E. foliata* samples generated paralog warnings for 124 exons on average, whereas 604 exons generated a warning for the average *E. fragilis* sample (three accessions). The mean sequence divergence based on all assembled sequences among *Ephedra* species is 2.2%, and 17.4% between *Ephedra* and *Welwitschia* and *Gnetum* individuals. The full assembly statistics can be found in Table S3. For practical reasons only loci that contained an outgroup sequence were used for downstream analyses, which resulted in 240 gene supercontig loci and 987 exon supercontig loci. Additional filtering steps will modify the number of exon supercontig loci for each dataset (Tables 1 and 2).

### 3.3 Dataset Characteristics

The 882 loci in the *paralog ortholog‐inferred* dataset (dataset 1) had an average locus length of 482 bp and a concatenated length of 424,791 bp (Table 1). A total of 44,513 sites were parsimony informative (PIS; 10%, mean 50 PIS per locus) for the *Ephedra* only data, and with the inclusion of outgroup sequences the number of PIS increased to 95,756 (23%) and a mean 109 PIS per locus (Table 1).

Selected dataset characteristics of the remaining datasets (datasets 2–6)—that is, number of loci, parsimony informative sites and average locus information, as well as average taxon occupancy—can be found in Table 1. For full alignment statistics of *Ephedra* across all datasets, including and excluding outgroups, see Table S4.

### 3.4 Species Tree Statistics

**FIGURE 2:**
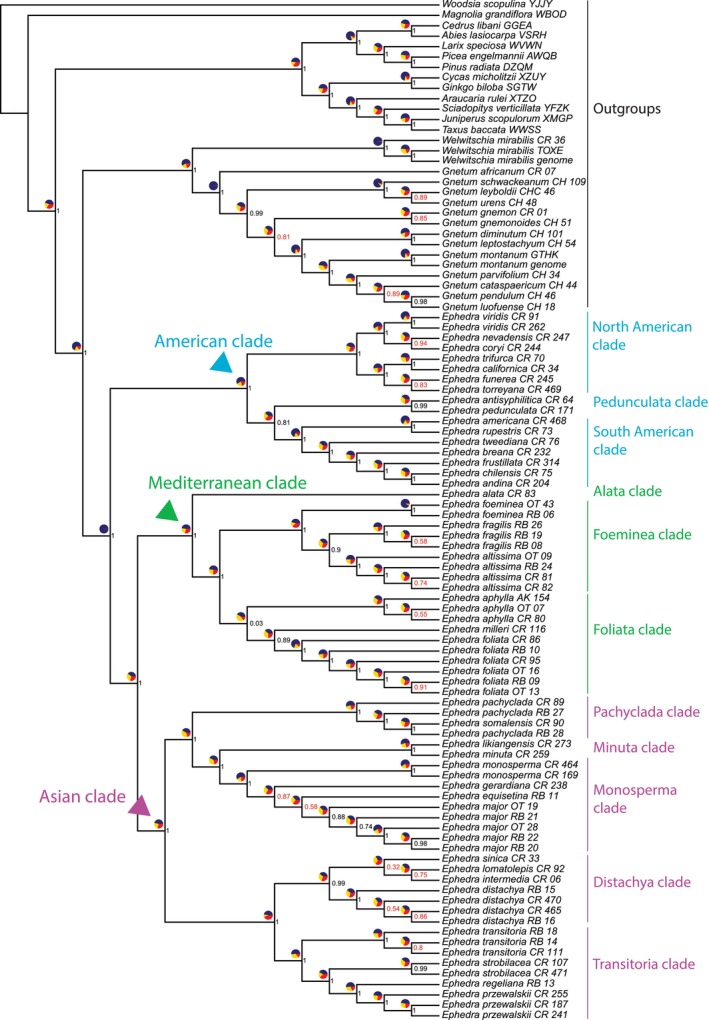
The species tree resulting from the ASTRAL‐III analysis of the *paralog ortholog‐inferred* dataset (dataset 1). Local posterior probabilities of clades are given (values in red indicate branches for which a polytomy could not be rejected). Pie charts indicate relative frequencies of alternative topologies around each branch (blue = congruent with species tree; yellow = first alternative topology; red = second alternative topology). Clade names are discussed in the text.

The species tree resulting from the ASTRAL analysis of the *paralog ortholog‐inferred* dataset (dataset 1, Figure 2) had a Normalized Quartet Score (NQS) of 0.71, and an average branch support (local posterior probability; LPP) of 0.94 (0.91 for ingroup results only) (Table 2). A polytomy could not be rejected for six branches in *Ephedra*, and for an additional four branches among the outgroups (Table 2). In the total phylogeny, 72 of the 97 branches had an LPP support of ≥ 95% (46 of 67 for the ingroup; Table 2).

**FIGURE 3:**
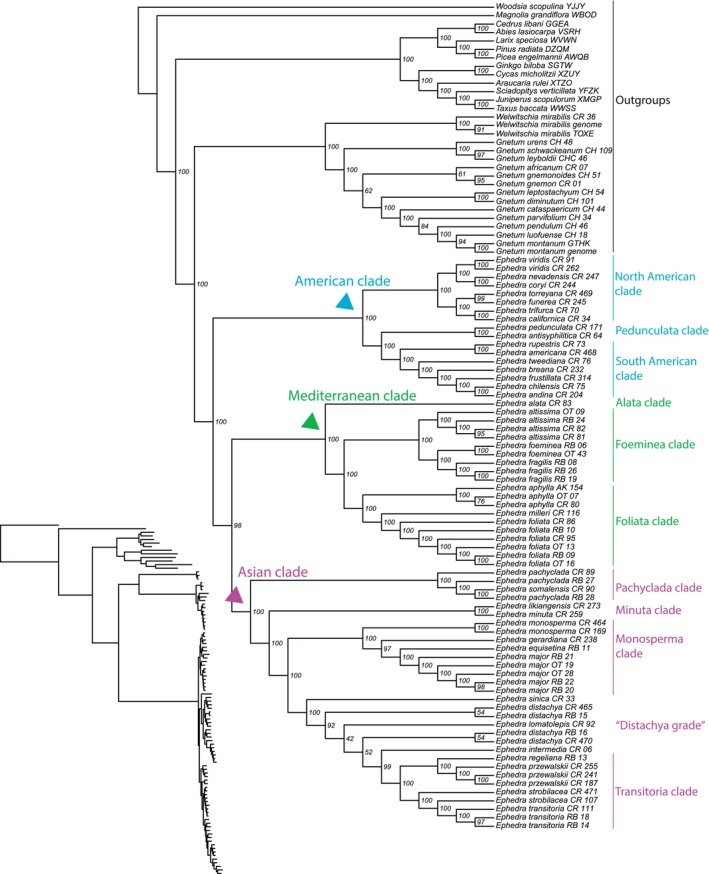
Phylogeny resulting from the maximum likelihood analysis of the concatenated version of the *paralog ortholog‐inferred* dataset (dataset 1). Bootstrap support is indicated for all branches. Phylogram to the left with branch lengths indicating the number of expected substitutions per site. Clade names are discussed in the text.

The phylogeny resulting from the maximum likelihood analysis of the *paralog ortholog‐inferred* dataset (dataset 1, Figure 3) had an average branch support (ML bootstrap; BS) of 0.93 (0.95 for ingroup results only) (Table 2). Eighty‐four of 97 branches (61 of 67 for the ingroup) had a BS support of ≥ 95% (Table 2).

An overview of characteristics of the remaining species trees (from datasets 2–6)—that is, branch support, gene tree discordance and ability to reject polytomies, as well as average bootstrap support for input gene trees for all datasets—are summarized in Table 2. Their respective topological results are shown in Figures S1–S5.

### 3.5 Putatively Paralogous Sequences

Both the ortholog inference strategy and the removal of loci with paralog warning flags strategy resulted in a reduction of loci included in the analysis with 11% and 88%, respectively. The strong reduction of loci in the *paralog 1to1 ortholog* dataset (dataset 2) is reflected back in the lower average branch support (LPP), fewer branches with an LPP support of ≥ 95%, and many branches that cannot be rejected as polytomies as compared to the remaining paralog handling strategies (datasets 1, 3–4, Table 2). The results of the ASTRAL analysis of the *paralog ortholog‐inferred* dataset (dataset 1) showed a lower average LPP branch support as compared to the paralog control dataset (*exon supercontig standard* dataset, dataset 4), but also less gene tree discordance as implied by the NQS (0.71 and 0.68, respectively) and fewer branches for which a polytomy could not be rejected (Table 2). Branch support values of the *paralog AstralPro* dataset (dataset 3) were relatively similar to those of the *paralog ortholog‐inferred* dataset (dataset 1) analyzed with ASTRAL (Table 2).

The largest phylogenetic distance, as measured with the Normalized Robinson‐Foulds (NRF) distance, was observed between the *paralog ortholog‐inferred* ASTRAL species tree (Figure 2) and the *paralog ortholog‐inferred* ML species tree (Figure 3) with an NRF of 0.22. The topological differences between these two species trees mainly concern the Distachya clade (comprising *E. sinica* , *E. intermedia* , *E. lomatolepis*, and *E. distachya* ), which has collapsed into a “grade” of successive sisters to the Transitoria clade (comprising *E. transitoria* , *E. strobilacea*, *E. regeliana*, and *E. przewalskii* ) in the results from the concatenated ML analysis (Figure 3). The fewest topological differences were observed between the *paralog ortholog‐inferred* ASTRAL species tree (Figure 2) and the *paralog AstralPro* species tree (Figure S2), with only the Distachya clade being in an alternative position within the Asian clade (NRF 0.08). All strategies we have used that somehow accommodated for potentially paralogous sequences resolved the American clade as sister to the remaining Old‐World species, whereas neglecting paralogous sequences resolved the Asian clade as sister to the remaining *Ephedra* species as indicated in the results of the paralog control tree (*exon supercontig standard* species tree, Figure S3). The Asia sister topology is when present always poorly supported, and a basal polytomy in *Ephedra* can never be rejected in these trees (Figures S3–S5). In contrast to all other analyses, the backbone phylogeny based on the ASTRAL analysis and the concatenated ML analysis of the *paralog ortholog‐inferred* dataset (dataset 1, Figures 2 and 3) is well supported, and the main topology is dominant over alternative topologies for all the deepest branches as assessed by the quartet frequencies of the ASTRAL output (Figure 2).

### 3.6 Recombinant Sequences

Significant evidence of recombination was detected in 92% of the loci that comprise the *gene supercontig* dataset (dataset 6) when employing the Phi Test. For the *exon supercontig standard* dataset (dataset 4), significant evidence of recombination was detected in 75% of the loci, representing 98% of the gene loci. Comparison between these two species trees: based on the *gene supercontig* dataset (dataset 6, Figure S5) that potentially may include recombinant sequences and the supposedly recombinant‐free *exon supercontig standard* dataset (dataset 4, Figure S3) showed several topological differences within the Mediterranean and Asian clades (NRF = 0.22). One of the differences concerns the Transitoria clade, which is monophyletic in the *exon supercontig standard* species tree (Figure S3) but polyphyletic in the *gene supercontig* species tree (Figure S5).

The species trees produced from the *exon supercontig standard* dataset (which includes loci with recombination as detected by the Phi Test, dataset 4, Figure S3) and the *exon supercontig recombination‐free* dataset (dataset 5, Figure S4) had relatively similar NQS values (0.68 vs. 0.67, respectively; Table 2). However, the *exon supercontig recombination‐free* species tree had lower average branch support, fewer branches with ≥ 0.95 branch support (LPP) and a considerably higher number of branches for which a polytomy could not be rejected compared to the *exon supercontig standard* species tree (Table 2). The NRF distance between the two species trees is 0.13, indicating some degree of conflict, which is mostly confined to the Mediterranean clade with both unsupported (e.g., the alternative positions of *E. aphylla* ) and supported (e.g., the respective positions of *E. alata* ) topological conflict.

### 3.7 Phylogeny of Ephedra

Across all phylogenetic trees *Ephedra* species are distributed in three geographically supported clades: the American clade, the Asian clade, and the Mediterranean clade (Figures 2 and 3; Figures S1–S5). Species trees from paralog‐handling strategies that utilized paralogous sequences all resolved an American clade sister relationship to the Old‐World clades regardless of analytical approach (Figures 2 and 3; Figures S1 and S2). The branch that separates the three geographical clades is however short (inset phylogram, Figure 3). In the results of all other approaches (Figures S3–S5), the well‐supported sister relationship between the American clade and the remaining genus (Figures 2 and 3) is replaced by an Asian clade sister relationship that is unsupported, and a basal polytomy can never be rejected in those trees.

Descriptions below of the relationships within the three geographically defined clades are based on the *paralog ortholog‐inferred* results depicted in Figures 2 and 3 given the curated nature of the dataset, the positive dataset statistics and the strongly supported topological backbone of the *Ephedra* phylogeny compared to those of other analytical approaches we employed. Key topological differences (if any) between these results and those of the ASTRAL‐Pro (Figure S2) and ASTRAL‐III analyses of the *exon supercontig standard* dataset and the *gene supercontig* dataset (Figures S3 and S5) are mentioned. The results of the strongly filtered (Figures S1 and S4) approaches are not considered as their severe reduction in loci number could have affected robust phylogenetic inference.

#### 3.7.1 American Clade

The relationships among American species were consistent and very well supported across the datasets (Figures 2 and 3; Figures S2, S3 and S5). Species comprising the American clade are distributed in two clades, which roughly follows a North American and a South American distribution; North American species are, however, not monophyletic (Figures 2 and 3; Figures S2, S3, and S5). While most North American species form a clade comprising two sister groups: *E. viridis* sister to *E. nevadensis* + *E. coryi* , and *E. trifurca* + *E. californica* sister to *E. funerea* + *E. torreyana*, the North American species *E. antisyphilitica* and *E. pedunculata* form a clade that is sister to the South American species (Figures 2 and 3; Figures S2, S3, and S5). Herein, *E. rupestris* and *E. americana* form a clade that is sister to the remaining South American species included here (*E. tweediana*, *E. breana*, *E. frustillata*, *E. chilensis* , *E. andina* ).

#### 3.7.2 Mediterranean Clade

*Ephedra alata* is well‐supported as sister to the remaining Mediterranean taxa (Figures 2 and 3; Figures S2, S3, and S5). The species *E. altissima* , *E. fragilis* , and *E. foeminea* form a clade (the Foeminea clade; Figures 2 and 3; Figures S2, S3, and S5) nested within a larger Mediterranean clade, but the relationship among these three species is unclear. *Ephedra aphylla, E. milleri*, and *E. foliata* form a clade where *E. aphylla* is sister to *E. milleri* and *E. foliata* (the Foliata clade, Figures 2 and 3; Figure S2). In two of the supplementary analyses, *Ephedra milleri* is instead sister to the other two, and *E. aphylla* is not monophyletic (Figures S3 and S5).

#### 3.7.3 Asian Clade

The Asian clade harbors many species that are distributed in five clades: the Pachyclada clade, the Minuta clade, the Monosperma clade, the Distachya clade, and the Transitoria clade (Figure 2; Figures S2 and S3). Relationships among and within these clades may, however, differ among results from different analytical approaches. For example, while the Pachyclada clade is always well supported and includes *E. somalensis* (Figures 2 and 3; Figures S2, S3, and S5), it may be strongly supported as sister to a clade comprising the Minuta clade and the Monosperma clade (Figure 2), to a clade comprising the Minuta clade, the Monosperma clade, and the Distachya clade (Figure S2), or to the remaining Asian species (Figure 3). The Minuta clade ( *E. minuta* and *E. likiangensis*) and the Monosperma clade (*E. monosperma, E. gerardiana, E. equisetina*, and *E. major* ) are also strongly supported in all analyses (Figures 2 and 3; Figures S2, S3, and S5). The Distachya clade ( *E. sinica* , *E. intermedia* , *E. lomatolepis*, and *E. distachya* ) is well supported in the coalescent‐based analysis of *paralog ortholog‐inferred* data (Figure 2) and present but less well supported in most supplementary coalescent‐based analyses (Figures S2 and S3), but the clade is collapsed into a “grade” in the concatenated ML analysis (Figure 3) and the *gene supercontig* tree (Figure S5). The Transitoria clade, comprising the species *E. transitoria* , *E. strobilacea*, *E. regeliana*, and *E. przewalskii*, is very well supported in most analyses (Figures 2 and 3; Figures S2 and S3), typically with *E. transitoria* sister to the remaining species in the clade (but see Figure 3). In the *gene supercontig* tree, the Transitoria clade is absent with *E. transitoria* resolved as sister to all other Asian species (Figure S5).

## 4 Discussion

Evolutionary relationships in *Ephedra* have been repeatedly investigated during more than two decades (Ickert‐Bond and Wojciechowski 2004; Rydin et al. 2004, 2010, 2021; Huang et al. 2005; Rydin and Korall 2009; Kakiuchi et al. 2011; Loera et al. 2015; Wu et al. 2016, 2021; Ickert‐Bond et al. 2020; Yu et al. 2023) and has with time proven to be a very difficult phylogenetic problem. The absence of a stable phylogenetic framework has hampered evolutionary studies (e.g., regarding biogeography, morphology, reproductive biology and ecology) in this ancient clade, documented in the fossil record at least since the latest Jurassic—Early Cretaceous (Rydin et al. 2004, 2021; Manchester et al. 2022; Barbolini et al. 2025). One reason for the problem is likely the low levels of sequence divergence among species of the genus, noted already in early studies based on molecular data (Ickert‐Bond and Wojciechowski 2004; Rydin et al. 2004; Huang et al. 2005; Rydin and Korall 2009). However, more recent work based on genomic data (Wu et al. 2016; Rydin et al. 2021; Wu et al. 2021 and the present study) indicates gene tree discordance as another important reason for the struggle to resolve the phylogeny of *Ephedra*.

Leveraging the information from flanking regions massively increased the amount of parsimony informative characters in the dataset and resulted in a well‐supported species phylogeny (Figure 2), which is largely congruent with that resulting from a maximum likelihood (ML) analysis of the same but concatenated dataset (Figure 3). We included this concatenated ML analysis because it can be preferable over species tree estimation using coalescent‐based summary methods when there is increased risk for gene tree error (as could be the case in *Ephedra* due to information poverty in individual loci). Removing the information from entire loci, for which sequence data are missing for some of the specimens in the dataset, has little support in the literature (Hosner et al. 2016; Molloy and Warnow 2018), but we used analytical tools to trim fragmentary data, prune away terminals on exceptionally long branches and collapse branches with a low BS support in the individual gene trees. We further investigated the potential effect of putative paralogy and recombination on phylogeny estimation in *Ephedra* and while both processes likely have limited influence on our results in general, some taxa in the Transitoria and Distachya clades did seem to be influenced by the presence of paralogs and/or intra‐locus recombination (further discussed below).

Our results strongly support the division of *Ephedra* species into three clades, which are geographically defined, with the American clade sister to the Mediterranean clade and the Asian clade (Figures 2 and 3). American species comprising the sister group to the remaining genus stands in sharp contrast to almost all results in the literature, in which the deepest splits in *Ephedra* have varied considerably among (and within) studies, but have typically indicated an origin of the genus in the Old World (Ickert‐Bond and Wojciechowski 2004; Rydin et al. 2004, 2010; Rydin and Korall 2009). Further, while relationships within the three major clades are consistent in most of our analyses, gene tree discordance can evidently be high throughout *Ephedra* (pie charts e.g., in Figure 2). To unambiguously explain the gene tree discordance observed in *Ephedra* based on our results is premature and gene tree estimation errors must also be considered; however, it appears plausible that both ILS and hybridization/introgression are important sources of topological conflict in *Ephedra*. Comparison of our *Ephedra* topology based on nearly a thousand nuclear exon loci with that obtained from plastome data in Rydin et al. (2021), suggests high levels of cytonuclear discordance within all the three geographically defined clades. It should be noted, however, that our taxon sample is not identical to that in Rydin et al. (2021). Additional studies are needed to investigate cytonuclear discordance and quantify the influence of ILS, hybridization and introgression as sources of topological conflict within *Ephedra*.

### 4.1 Putatively Paralogous Sequences

Putatively paralogous sequences (as indicated by output from HybPiper) are present in abundance in the exon‐based datasets and follow a seemingly species‐specific distribution. Observations of gene trees built with putatively paralogous sequences included besides orthologous sequences showed gene trees where gene copies grouped together, which agrees with patterns of true paralogous sequences. Paralogous sequences are expected in the datasets given the abundance of polyploid individuals in *Ephedra* (Ickert‐Bond et al. 2020), and thus it seems plausible that the gross of putatively paralogous sequences in *Ephedra* reflects true paralogous sequences. However, paralogous sequences are unwanted during phylogenetic reconstruction as they could seriously mislead phylogenetic inference unless accounted for.

Fortunately, handling of paralogous sequences can be done in several ways (Ufimov et al. 2022; Joyce et al. 2025). One approach is to simply remove loci with putatively paralogous sequences from the analysis (see e.g., Kuhnhäuser et al. 2021; Nge et al. 2024). Applying a strict one‐to‐one ortholog approach to the *Ephedra* dataset by removing all loci that contained putatively paralog sequences resulted in a loss of 88% of the loci. The impact of removing loci with paralog warnings mainly affected the branch support values negatively, a statistic known to be influenced by the number of loci utilized (Sayyari and Mirarab 2016). Extensive loci removal does not always affect topology and statistics in species tree estimation (Thureborn et al. 2022), but for a group like *Ephedra*, known to have undergone whole genome duplication and having limited sequence divergence among species, such an approach can be particularly unfortunate. Furthermore, paralogous sequences in the form of pseudo‐orthologs or artefactual orthologs are not removed with a one‐to‐one approach and might still be present in the remaining loci (Smith and Hahn 2021; Frost et al. 2024).

On the other hand, paralogous sequences can provide valuable information, for example, they double the information of speciation events that occurred after duplication events, which can be particularly useful when gene copies have been lost (Smith and Hahn 2021). Allowing paralogous sequences to be part of the phylogenetic reconstruction workflows in *Ephedra*, as we did for our *paralog ortholog‐inferred* dataset (dataset 1, Figures 2 and 3) and the *paralog AstralPro* dataset (dataset 3, Figure S2), yielded robust results (Table 2) and well‐supported topologies, which were largely similar and differed only at a few branches (Figures 2 and 3; Figure S2). In fact, ortholog detection from gene trees built with putatively paralogous‐ and orthologous sequences, analyzed with ASTRAL, resolved the species tree with the highest quartet score (indicating less gene tree discordance) and the fewest branches for which a polytomy could not be rejected as compared to those of all other paralog handling approaches (Table 2). We thus agree with the notion expressed in several studies in the literature that for biological datasets a curated approach to paralogs in the data might be beneficial for phylogenetic reconstruction (Siu‐Ting et al. 2019; Gardner et al. 2021; Morales‐Briones et al. 2022; Frost et al. 2024).

Analysis of the paralog control dataset (*exon supercontig standard* dataset, dataset 4) where paralog warnings were ignored yielded a species tree (Figure S3) that was largely similar in topology and branch support compared to those of other paralog handling approaches. However, paralog sequences will (when ignored) increase gene tree discordance, a consequence observed in our study. The normalized quartet score (NQS) is lower for the results of the paralog control dataset compared with that of the *paralog ortholog‐inferred* dataset analyzed with ASTRAL (NQS = 0.68 vs. 0.71; Table 2). Thus, even though studies have indicated that the summary method ASTRAL‐III is relatively robust to the presence of paralogs (Yan et al. 2022), handling putative paralogs does improve species tree estimation in *Ephedra*.

The strong similarity in topology, the relatively similar NQS and branch statistics in the results of our paralog handling approaches suggest, however, that (putatively) paralogous sequences are likely not a main source of conflict in *Ephedra*. Ickert‐Bond et al. (2020) argued that the polyploid nature of *Ephedra* species is a relatively recent occurrence, which would mean that true paralogous sequences in *Ephedra* have likely not accumulated enough mutations to be able to seriously mislead phylogenetic reconstruction, a conclusion our results support. The few topological differences that do exist among our results from different handling of putative paralogs are perhaps better explained by homoeologous sequences resulting from the allopolyploid nature of some of the taxa in the conflicting clades, such as *E. intermedia* , an established allopolyploid (Wu et al. 2021), instead of true paralogous sequences alone.

### 4.2 Recombinant Sequences

Intra‐locus recombination was detected in 94% and 69% of the loci that constitute the gene‐ and exon supercontig datasets, respectively, which indicates that genetic recombination within loci might be a common feature in *Ephedra* that cannot be escaped in species tree estimation by breaking up the gene loci into “shorter” (exon) loci as has sometimes been suggested (Gatesy and Springer 2014). The presence of intra‐locus recombination in a dataset is unwanted in analyses that employ the multispecies coalescent model (MSCM), as the model assumes that recombination occurs freely between loci but is absent within loci. On the other hand, studies have shown that species tree estimation under the MSCM is largely robust to the presence of recombination in a dataset (Lanier and Knowles 2012; Wang and Liu 2016). Running the MSCM on the exon supercontig dataset with presumably recombinant‐free loci (dataset 5, Table 2, Figure S4) yielded a different topology compared to that of the *exon supercontig standard* dataset (dataset 4, Table 2, Figure S3); however, the lower overall support for the former species tree leaves the topological result uncertain. Gene tree discordance (as assessed by NQS values) in the two species trees is roughly the same, 0.67 vs. 0.68, which suggests that although intra‐locus recombination occurs relatively frequently in *Ephedra*, it does not hamper species tree estimation, which would agree with conclusions in studies in the literature that evaluated recombinational influence on their species tree estimation (Folk et al. 2017; Morales‐Briones et al. 2018).

A stitched (chimeric) sequence is also a recombinant sequence, albeit artificial. Comparing the results based on the *gene supercontig* dataset (dataset 6, Table 2, Figure S5) with those from the *exon supercontig standard* dataset (dataset 4, Table 2, Figure S3), that is, presumably stitched vs. non‐stitched sequences, respectively, showed phylogenetically well resolved species trees with a few topological differences. It is difficult to fully compare the results of the *gene supercontig* dataset (Figure S5) with those of the *exon supercontig standard* dataset (Figure S3) since the statistic output gives mixed impressions (Table 2). The number of loci is much fewer in the gene dataset although the amount of information in each locus is conceivably larger, which may bias comparison of statistical output. The main topological differences between these two species trees are that the Distachya clade is paraphyletic and the Transitoria clade is polyphyletic in the results based on the *gene supercontig* dataset (Figure S5), a result not seen in any other species tree (but note the Distachya “grade” in the ML‐based analysis, Figure 3). Some taxa in these clades are known to have undergone allopolyploidy (Wu et al. 2021), and we conclude that although the *gene supercontig* dataset conceivably contains stitched sequences, their problematic influence on phylogenetic reconstruction appears limited to taxa with a previously documented history of allopolyploidy.

### 4.3 The Ephedra Phylogeny

Results from phylogeny estimations in *Ephedra* have varied considerably in the literature. Often, an individual taxon from eastern Europe to western‐central Asia was placed as sister to the remaining *Ephedra* species (e.g., *E. laristanica* [= *E. foliata* ] in Ickert‐Bond and Wojciechowski (2004); *E. foeminea* in Rydin and Korall (2009) and *E. major* in Rydin et al. (2021)), rendering geographically defined Old‐World clade(s) paraphyletic. Albeit the results were typically unsupported and sometimes only a matter of rooting option; the consequence has been a knowledge gap regarding relationships in *Ephedra*, including its relationship to the extensive fossil diversity of the group (see e.g., Rydin et al. 2010). The root of the crown group and its age have been notorious difficult to establish and seems to be affected by factors like the choice of (molecular) markers and rooting strategy (Rydin et al. 2021). Our results show that biological processes such as ILS and/or horizontal transfer of genetic material also could have contributed to the uncertainty regarding the deepest splits in *Ephedra*.

For a long time, *E*. *foeminea* was considered the sister lineage to the remaining *Ephedra* species, a result that was established based on data from several chloroplast and nuclear ribosomal markers (Rydin and Korall 2009) and supported by reproductive characters and processes (Bolinder, Humphreys, et al. 2016). However, analyses of the entire chloroplast genome as well as the nuclear ribosomal cistron contested the sister position of *E. foeminea* (Rydin et al. 2021), and results of the present study confirm that conclusion. Here, *E. foeminea* forms a clade together with *E. fragilis* and *E. altissima*, and this clade is nested within a larger Mediterranean clade (Figures 2 and 3; Figures S2, S3, and S5). The distribution of *Ephedra* species into three well‐supported and geographically defined clades (the American clade, the Asian clade and the Mediterranean clade) has been found in one previous study, which analyzed plastome data in a Bayesian framework using a relaxed molecular clock rooting approach (Rydin et al. 2021), but interrelationships among the three clades were different than in the present study. The sister‐relationship of the American clade to the remaining genus is well supported in our ASTRAL and concatenated ML analyses of the *paralog ortholog‐inferred* data (Figures 2 and 3), and present but unsupported in the remaining paralog‐based trees (Figures S1 and S2), but the quartet frequencies associated with the branch uniting the Mediterranean and the Asian clades are nearly equal, which indicates strong gene tree discordance. Therefore, the sister placement of the American clade should be interpreted with caution.

#### 4.3.1 The American Clade

Previous work has typically demonstrated monophyly of the American clade (Ickert‐Bond and Wojciechowski 2004; Rydin et al. 2004, 2021; Huang et al. 2005; Rydin and Korall 2009), but results among species have remained uncertain. Our analyses yielded consistent and very well‐supported results with no topological conflict (Figures 2 and 3; Figures S2, S3, and S5). The results support the previous division of American *Ephedra* species into two clades: one comprises most of the North American species, whereas the other encompasses the South American species sister to the two North American species, *E. pedunculata* and *E. antisyphilitica* . As shown elsewhere (e.g., Ickert‐Bond and Wojciechowski 2004; Loera et al. 2015), both the North American clade and the South American clade include ample additional species diversity not analyzed here, and further studies are warranted.

##### 4.3.1.1 The (main) North American clade

Phylogenetic relationships among North American species have often remained unresolved or at best partly resolved in analyses based on information from the entire nuclear ribosomal cistron or smaller parts thereof (Ickert‐Bond and Wojciechowski 2004; Rydin and Korall 2009; Rydin et al. 2010, 2021). This is probably best reflected in the species complex involving the taxa *E. torreyana* , *E. funerea* , *E. nevadensis* , *E. antisyphilitica* , and *E. coryi* , where these taxa often formed a soft polytomy. Here, utilizing the information present in hundreds of nuclear exons and their non‐coding flanking regions resulted in a robust phylogeny of the North American taxa. A notable difference compared to previous studies using nuclear (ribosomal) data is the position of *E. viridis* as sister to *E. nevadensis* and *E. coryi* , but also the non‐sister relationship between *E. coryi* and *E. antisyphilitica* , of which the latter has even been placed outside of the larger North American clade in the present study.

Despite the good resolution among the species in the main North American clade, a clear picture of their evolution remains to be established. Plastome data support alternative relationships, indicating cytonuclear discordance. Ickert‐Bond et al. (2020) showed that all North American species (except *E. compacta* ) are polyploids or have polyploid cytotypes, and there are reports of hybridization (Cutler 1939; Wendt 1993), which likely has contributed to the previous uncertainty and now clearly documented cytonuclear discordance revolving around the phylogeny of North American taxa.

##### 4.3.1.2 The Pedunculata clade

*Ephedra antisyphilitica* and *E. pedunculata* are strongly supported as sisters, and sister to the South American clade. This is surprising since analyses based on nuclear ribosomal data have almost always placed *E. antisyphilitica* as sister to *E. coryi* (Ickert‐Bond and Wojciechowski 2004; Rydin and Korall 2009; Rydin et al. 2010; Loera et al. 2015). On the other hand, plastome data resolved all three species as a clade sister to South American species (Rydin et al. 2021). A close relationship between the three species would be supported by morphological and biogeographical information as they all have fleshy bracts surrounding their seeds at maturity, and can be found in the state of Texas and in North Eastern Mexico, respectively, whereas other North American species have dry, papery or membranous bracts and inhabit deserts mainly in South‐Western United States and North‐Western Mexico (Stevenson 1993). Additional data and analyses are however needed to assess the cytonuclear discordance regarding the phylogenetic position of *E. coryi* .

The enigmatic position of *E. pedunculata* appears here to be resolved. It has previously been sister to all American species or sister to the South American species, albeit together with one or multiple other North American species (Ickert‐Bond and Wojciechowski 2004; Rydin and Korall 2009; Loera et al. 2015; Rydin et al. 2021). Based on the same sample data from the plastome and the nuclear ribosomal cistron, Rydin et al. (2021) argued that the apparent cytonuclear discordance regarding the placement of *E. pedunculata* could be due to a history of hybridization and/or introgression or an allopolyploid origin of the species. However, our low‐copy nuclear loci support a relationship between *E. pedunculata* and *E. antisyphilitica*, this clade being sister to the South American clade, as does the plastome (with the inclusion of *E. coryi* in the *E. pedunculata* — *E. antisyphilitica* clade) (Rydin et al. 2021), thus refuting cytonuclear discordance regarding the position of *E. pedunculata* .

##### 4.3.1.3 The South American Clade

Phylogenetic reconstruction of South American species shows strong resemblance to that of the North American species in that nuclear ribosomal data used in previous work contained limited information (Ickert‐Bond and Wojciechowski 2004; Rydin and Korall 2009; Rydin et al. 2010). Here, the South American species form a well resolved monophyletic group with *E. americana* and *E. rupestris* as sister taxa forming the sister clade to the remaining South American species. This result is congruent with results based on plastome data (Rydin et al. 2021), whereas our results among the remaining South American species differ from those obtained based on plastome data (Rydin et al. 2021). As the North American clade, the South American clade contains additional diversity (see e.g., Ickert‐Bond and Wojciechowski 2004; Loera et al. 2015) not sampled here, and a more complete species sampling and inclusion of multiple accession for individual species would be warranted.

#### 4.3.2 The Mediterranean Clade

Our analyses based on low‐copy nuclear information support a monophyletic group of species distributed in the area surrounding the Mediterranean Sea. Most of the species are distributed into two clades, with *E. alata* being their sister lineage (Figures 2 and 3; Figures S2, S3, and S5). These results stand in sharp contrast with those of previous work based on plastid and nuclear ribosomal data (Rydin and Korall 2009; Rydin et al. 2010, 2021). Further investigations of species delimitations and evolutionary relationships among the Mediterranean diversity of *Ephedra* based on dense samples of taxa and data are needed.

##### 4.3.2.1 The Alata Clade

In all our analyses, *E. alata* is strongly supported as sister to the remaining species in the Mediterranean clade (but see Figure S4). Nuclear information has often placed *E. alata* as an independent lineage or as sister to *E. milleri* within a larger, mainly unresolved Mediterranean clade (Rydin and Korall 2009; Rydin et al. 2010, 2021; Ickert‐Bond et al. 2020). That *E. alata* separates from other Mediterranean species is not surprising as it is the only Mediterranean species that has dry, papery bracts surrounding the seeds at maturation. Evolutionary shifts in seed dispersal mechanism appear to have occurred several times in *Ephedra* (Hollander et al. 2010).

##### 4.3.2.2 The Foeminea Clade

A Mediterranean clade comprising *E. foeminea*, *E. fragilis* and *E. altissima* is strongly supported in nearly all our trees, although the interrelationships of these species vary among results (e.g., Figures 2 and 3). Previous studies that included a comprehensive sampling of Mediterranean species and utilized molecular data have not placed *E. foeminea, E. fragilis* and *E. altissima* in a clade that is distinct from other species (Rydin and Korall 2009; Rydin et al. 2010, 2021). A close affinity between *E. altissima* and *E. fragilis*, as indicated in some trees (Figure 2; Figures S2 and S5) is supported by a common trait that is basically unique for the two species: the presence of undifferentiated parenchymatous tissue and scattered fibers in the mesophyll layer of the seed envelope (Rydin et al. 2010). Further, both species are native to the Atlas Mountain (Desfontaines 1798). These features do not hold for *E. foeminea*, which has a more eastern Mediterranean distribution and the mesophyll of its seed envelope is differentiate into two distinct zones (Rydin et al. 2010). In some other trees (Figures 3 and S3), *E. foeminea* and *E. fragilis* are sisters instead. These species are united by the presence of insect pollination (also suggested for *E. aphylla* ; (Meeuse et al. 1990)); however, extensive studies have shown that the dependency on animals for pollination varies between these species. While *E. foeminea* is entirely dependent on insect pollinators for survival (Bolinder, Humphreys, et al. 2016), *E. fragilis* is not. Instead, lizards and insects provide merely a complementary contribution in addition to wind pollination in *E. fragilis* (Celedón‐Neghme et al. 2016; Fuster and Traveset 2019). It is further interesting to note that over a century ago, *E. foeminea* was considered to be a variety of *E. fragilis* , under the former name *E. campylopoda* C.A.Mey., as *E. fragilis* var. *campylopoda* (C.A.Mey.) Stapf, because of seemingly little variation between *E. campylopoda* and *E. fragilis* to acknowledge a species status for the former (Stapf 1889; Freitag and Maier‐Stolte 1989). Nowadays the species status of *E. foeminea* is not questioned and our results support *E. foeminea* as a robust species.

##### 4.3.2.3 The Foliata Clade

*Ephedra aphylla* has often been included in a clade together with *E. fragilis* and *E. altissima* in previous work (Rydin and Korall 2009; Rydin et al. 2010, 2021). Here, *E. aphylla* is instead resolved in a clade together with *E. foliata* and *E. milleri* (Figures 2 and 3; Figures S2 and S5), however, one supplementary analysis does not support monophyly of *E. aphylla* (Figure S3). Based on the uneven quartet frequencies of the second and third alternative topologies (e.g., in Figure 2), it seems plausible that hybridization and/or introgression have played a role in the deeper splits of the Foliata clade. A hybrid status has previously been discussed for *E. milleri*, with the suggested putative parental species being *E. alata* (based on nuclear ribosomal data), and a species of the clade comprising *E. distachya* , *E. transitoria* and *E. pachyclada* (based on chloroplast data) (Rydin et al. 2021). Our results instead indicate that *E. aphylla* or *E. foliata*, both with distributions that partly overlap with several species of the Asian clade, could be the Mediterranean parent of *E. milleri*. Based on morphology, *E. milleri* could be a hybrid between *E. foliata* and *E. pachyclada* . *Ephedra milleri* shares several morphological and structural similarities with *E. foliata* (such as reproductive structures and bracts) and *E. pachyclada* (such as growth form and color of branches), and their distributions overlap at the southern end of the Arabian Peninsula, in Oman, the area where *E. milleri* was discovered and to which it as far as known is endemic (Freitag and Maier‐Stolte 1992).

The position of *E. foliata* has varied significantly in past work based on molecular data. In older studies, *E. foliata* was sister to all remaining *Ephedra* species (sometimes under the synonymous name *E. laristanica*) (Ickert‐Bond and Wojciechowski 2004; Rydin et al. 2021), or sister to the American and Asian clades (Rydin and Korall 2009; Rydin et al. 2010), or nested within a larger Mediterranean clade based on plastome data (Rydin et al. 2021). *Ephedra foliata* is nested in a larger Mediterranean clade also in the present study, but a Foliata clade comprising *E. foliata, E. aphylla*, and *E. milleri* (Figures 2 and 3; Figures S2 and S5) has not been present in any previous study.

#### 4.3.3 The Asian Clade

The species of the Asian clade are distributed in five strongly supported subclades (the Pachyclada clade, the Minuta clade, the Monosperma clade, the Distachya clade and the Transitoria clade [Figure 2; Figures S2 and S3]). In the concatenation analysis of the *paralog ortholog‐inferred* dataset and one supplementary analysis, the Distachya clade is collapsed into a “grade” of successive sisters to the Transitoria clade (Figures 3 and S5). This latter result is congruent with the results based on nuclear ribosomal data in previous work (Rydin and Korall 2009; Rydin et al. 2010, 2021). However, results among and within these four‐five clades have varied considerably in previous work as well as in the present study. In the species tree estimation based on the *paralog ortholog‐inferred* dataset with ASTRAL, the Asian clade is divided into two main clades: a Pachyclada‐Minuta‐Monosperma clade and a Distachya‐Transitoria clade (Figure 2). In the concatenated analysis of the same data, the Pachyclada clade is sister to the remaining Asian species (Figure 3). In contrast with most studies in the literature, our results are very strongly supported (Figures 2 and 3) and in the species tree, the main topology is clearly dominant over alternative topologies for all the Asian subclades except the Distachya clade (Figure 2).

Clearly, however, gene tree discordance is high for many branches in the Asian clade (as well as in the American and Mediterranean clades; Figure 2). Studies addressing phylogenetic discordance in *Ephedra* have been performed for some Asian taxa, in particular those that inhabit and neighbor the Qinghai Tibetan Plateau (Qin et al. 2013; Wu et al. 2016, 2021). As in the remaining *Ephedra* (Ickert‐Bond et al. 2020), many species are tetraploid (e.g., *E. intermedia, E. likiangensis* and *E. sinica* ) and Wu et al. (2016) argued that they are of allopolyploid origin. For other species (e.g., *E. gerardiana, E. regeliana* and *E. przewalskii* ) both tetraploid and diploid individuals are detected (Wu et al. 2016). It is, however, difficult to draw general conclusions on hybridization based on ploidy levels alone and it is clear that evolution in the Asian clade is only partly understood and needs more research.

##### 4.3.3.1 The Pachyclada Clade

The Pachyclada clade comprises *E. pachyclada* and *E. somalensis* . As shown previously (e.g., Rydin and Korall 2009) *E. somalensis* is nested among specimens of *E. pachyclada* , and the two taxa should probably be considered synonymous if these results persist in work with extended sampling in the clade. Results in Rydin et al. (2021) strongly indicate cytonuclear discordance regarding the position of the Pachyclada clade, something that is not contradicted here. Our results differ considerably from those based on plastome data (Rydin et al. 2021).

##### 4.3.3.2 The Minuta Clade

The Minuta clade comprises *E. likiangensis* and *E. minuta* . In previous work based on nuclear ribosomal data, this well‐supported clade has been sister to the remaining Asian species (Rydin and Korall 2009; Rydin et al. 2010) or to the Asian + American clades (Rydin et al. 2021). Here, its position in the Asian clade varies (e.g., Figures 2 and 3) and it should be noted that results in Rydin et al. (2021) revealed cytonuclear discordance regarding *E. likiangensis* and *E. minuta* in their study based on plastome data and nuclear ribosomal cistron data from the same samples. While the two species were strongly supported as sisters in the tree based on nuclear data, congruent with results in earlier studies, they were not sisters and nested among some species of the Monosperma clade in the well‐resolved and supported tree based on plastome data (Rydin et al. 2021).

##### 4.3.3.3 The Monosperma Clade

The Monosperma clade comprises the species *E. monosperma*, *E. gerardiana* , *E. equisetina* and *E. major* . Previous work (Rydin and Korall 2009; Rydin et al. 2010; Kakiuchi et al. 2011) indicates that the clade includes additional diversity (*E. rhytidosperma, E* *. saxatilis* ). The clade is well supported and *E. monosperma* is sister to the other species included here, followed by *E. gerardiana* sister to an *E. equisetina* — *E. major* clade (Figures 2 and 3; Figures S2, S3, and S5). The results are mostly well supported but the high levels of ILS indicated by quartet frequencies are detected for most branches (pie charts e.g., in Figure 2) and it can be noted that Freitag and Maier‐Stolte (1994) considered the latter three species synonymous. Further, from comparison with plastome‐based results in Rydin et al. (2021), cytonuclear discordance is evident among species of the Monosperma clade. Wu et al. (2021) argued that allopolyploidy has played an important role in the evolution in the Asian clade, and they argue for example that *E. monosperma* and *E. equisetina* contributed to the tetraploid genomes of *E. sinica* and *E. intermedia* . As for most subclades of *Ephedra*, further studies are necessary to elucidate reticulate evolution and polyploidy in the Monosperma clade.

##### 4.3.3.4 The Distachya + Transitoria Clade

The remaining species of the Asian clade sampled in the present study are included in a large clade (Figures 2 and 3), which in the coalescent‐based results of the *paralog ortholog‐inferred* dataset (Figure 2) is divided into a Distachya clade ( *E. sinica* , *E. intermedia* , *E. lomatolepis*, and *E. distachya* ), and a Transitoria clade ( *E. transitoria* , *E. strobilacea*, *E. regeliana*, *E. przewalskii* ). Gene tree discordance is high for many branches, which is apparent in the Distachya clade and could be caused by various factors, for example, ILS and/or hybridization/introgression (Figure 2). Furthermore, recent studies based on large amounts of genomic data (Rydin et al. 2021) demonstrate results within the Distachya+Transitoria clade that deviate substantially from those of the present study. Comparison with results in other previous studies (Ickert‐Bond and Wojciechowski 2004; Rydin and Korall 2009; Kakiuchi et al. 2011; Rydin et al. 2021) is complicated by different or limited taxon sampling and/or limited amount of sequence data in older work, resulting in poor resolution and statistic support. Nevertheless, conflicting results are evident; many previously established relationships are not recovered here.

##### 4.3.3.5 The Transitoria clade

*Ephedra transitoria, E. sarcocarpa* and *E. strobilacea* formed a well‐supported clade in several previous studies (Rydin and Korall 2009; Rydin et al. 2010; Kakiuchi et al. 2011). The species are morphologically similar and inhabit the arid semideserts of the Irano‐Thuranian region or parts thereof (Freitag and Maier‐Stolte 1994). Here *E. transitoria* and *E. strobilacea* are resolved together with *E. regeliana* and *E. przewalskii* in a very well supported clade (Figures 2 and 3). The inclusion of the latter two species is noteworthy, since they often formed a clade together with *E. sinica* , *E. intermedia* and *E. lomatolepis* in previous studies (Rydin and Korall 2009; Kakiuchi et al. 2011). It can further be noted that the position of *E. transitoria* may vary among our results although it is typically sister to the remaining species in the Transitoria clade (Figure 2; Figures S2 and S3).

## 5 Conclusions

Ever since the first phylogenetic studies of *Ephedra* using modern analytical tools were conducted more than 20 years ago (Rydin et al. 2002, 2004; Ickert‐Bond and Wojciechowski 2004), the difficulty in resolving species relationships in the group has been evident. Resolution was often incomplete in those studies, and statistical uncertainty has been the rule rather than the exception. The difficulty has not been confined to resolving the phylogeny, but has in addition concerned relationships among extant and extinct species (Rydin et al. 2004, 2006), the evolution of morphological traits through time (Rydin et al. 2010), pollen morphology (Bolinder, Norbäck Ivarsson, et al. 2016), and pollination syndromes (Bolinder, Humphreys, et al. 2016; Barbolini et al. 2025), and node age estimation and biogeography (Rydin et al. 2021).

It is difficult to assess how to compare phylogenetic results from older studies based on a handful of markers with those of the present study. For example, the total concatenated length of the dataset used in Rydin and Korall (2009) was 8077 bp, of which the vast majority of the (limited) information came from nuclear ribosomal ITS. The datasets used here are massively larger (e.g., 424,791 bp in dataset 1, of which 44,513 bp are parsimony informative within *Ephedra*; Table 1). It is however clear that the results of the present study manifest that evolution in *Ephedra* is an intractable problem that continues to elude science. While statistic support for our results is generally very strong (Figures 2 and 3; more easily obtained in work based larger amounts of data), it is equally clear that gene tree discordance is generally high. One of the most striking examples is the deepest divergence in the genus and the similar quartet frequencies at the branch uniting the Mediterranean and Asian clades (Figure 2). Gene tree discordance can be derived from gene tree estimation error (in addition to “real” biological reasons), something we have strived to minimize the risk for by including information from non‐coding data, employing data trimming and pruning of extreme branches and fragmentary sequences, collapsing branches with low support, and orthology inference in gene trees.

Putatively paralogous sequences and recombinant sequences (biological and/or artificial) seemed to have limited influence on phylogeny reconstruction in *Ephedra*. Even though handling putatively paralogous sequences and recombinant sequences did improve phylogenetic resolution and species tree statistics, in particular in clades that include known allopolyploids, we find that they should not be considered main sources of gene tree discordance in *Ephedra*. Instead, ILS and hybridization/introgression are expected to be common, although their extent and relative impact cannot be firmly concluded based on our results alone. Additional investigations of cytonuclear discordance, preferably using a same‐sample approach, would be worthwhile in order to address hypotheses of hybridization/introgression and processes such as chloroplast capture. Furthermore, in‐depth investigations of subclades of *Ephedra*, assessing species delimitations and phylogeny using massively increased sampling of specimens would be highly valuable.

Thus, while the three geographically defined clades of *Ephedra*, the American clade, the Mediterranean clade and the Asian clade, are firmly established and understanding of relationships within them has and can continue to be improved, the interrelationship of these three major clades is unclear and may very well remain so. This situation is not unique to *Ephedra* but has been indicated for several other organism groups (see e.g., discussions in Morales‐Briones et al. 2022). A major reason often attributed as cause for high levels of gene tree discordance is rapid evolution (Degnan and Rosenberg 2009), which seems true for the early evolution of the crown group *Ephedra* considering that the deepest branches are evidently short (inset phylogram, Figure 3). To trace such rapid radiation can be difficult also with extensive data sampling since the amount of information can nevertheless be low, in particular in individual loci, and may render sensitivity to model selection and inference methods (Mclean et al. 2019). Based on our results, all these issues clearly apply to *Ephedra*.

## Author Contributions

**Ruben Blokzijl:** conceptualization (equal), data curation (lead), formal analysis (lead), investigation (lead), validation (equal), visualization (equal), writing – original draft (lead), writing – review and editing (equal). **Catarina Rydin:** conceptualization (equal), funding acquisition (lead), investigation (supporting), project administration (lead), resources (lead), supervision (lead), validation (equal), visualization (equal), writing – original draft (supporting), writing – review and editing (equal).

## Funding

This work was supported by Vetenskapsrådet, 2017‐03985.

## Conflicts of Interest

The authors declare no conflicts of interest.

## Supporting information


**Table S1:** Taxon name, distribution and voucher information, and the European Nucleotide Archive (ENA) accession number for each sample used in this study.

**Table S2:** Raw read statistics showing the number of reads before, during and after deduplication, and quality trimming for the samples used in this study.

**Table S3:** Assembly statistics for the three datatypes assembled in this study, including the percentage of filtered reads on target, the number of genes with assembled contigs and paralog warnings.

**Table S4:** Full statistic report of all the datasets separated into an *Ephedra* only and an *Ephedra* and outgroup version, including taxon coverage, alignment length and informative sites.

**Figure S1:** The species tree resulting from the ASTRAL‐III analysis of the restricted ortholog only dataset (*paralog 1to1 ortholog* dataset, dataset 2) where all loci with putative paralog warnings were excluded. Local posterior probabilities of clades are given (values in red indicate branches for which a polytomy could not be rejected). Pie charts indicate relative frequencies of alternative topologies around each branch (blue = congruent with species tree; yellow = first alternative topology; red = second alternative topology). Clade names are discussed in the text.
**Figure S2:** The species tree resulting from the ASTRAL‐Pro analysis of the *paralog AstralPro* dataset (dataset 3). Local posterior probabilities of clades are given. Pie charts indicate relative frequencies of alternative topologies around each branch (blue = congruent with species tree; yellow = first alternative topology; red = second alternative topology). Clade names are discussed in the text.
**Figure S3:** The species tree resulting from the ASTRAL‐III analysis of the *exon supercontig standard* dataset (dataset 4) with exons + regions flanking the exons from a single sequence per accession (i.e., the standard output from HybPiper). Local posterior probabilities of clades are given (values in red indicate branches for which a polytomy could not be rejected). Pie charts indicate relative frequencies of alternative topologies around each branch (blue = congruent with species tree; yellow = first alternative topology; red = second alternative topology). Clade names are discussed in the text.
**Figure S4:** The species tree resulting from the ASTRAL‐III analysis of the *exon supercontig recombination‐free* dataset (dataset 5) where loci with evidence for recombination were removed. Local posterior probabilities of clades are given (values in red indicate branches for which a polytomy could not be rejected). Pie charts indicate relative frequencies of alternative topologies around each branch (blue = congruent with species tree; yellow = first alternative topology; red = second alternative topology). Clade names are discussed in the text.
**Figure S5:** The species tree resulting from the ASTRAL‐III analysis of the *gene supercontig* dataset (dataset 6) with exons, introns + regions flanking the gene. Local posterior probabilities of clades are given (values in red indicate branches for which a polytomy could not be rejected). Pie charts indicate relative frequencies of alternative topologies around each branch (blue = congruent with species tree; yellow = first alternative topology; red = second alternative topology). Clade names are discussed in the text.

## Data Availability

Raw sequence reads generated for the present study are deposited in the European Nucleotide Archive (ENA) under projects PRJEB89429 and PRJEB107040. The ENA accession numbers of the samples used are given in Table S1. The target files for bait design and assembly, as well as the assembled sequences for each dataset used in the study, are available from the Dryad digital repository (doi: 10.5061/dryad.m63xsj4g4).
