# Supplementary material for: Using Nuclear Genomic Data to Address Intractable Relationships and Gene Tree Discordance in an Ancient Group of Gymnosperms (Ephedra, Gnetales)
Source: Ecol Evol. 2026 Jun 30;16(7):e73863. doi: 10.1002/ece3.73863 (PMC13318514; doi:10.1002/ece3.73863)
Supplement: Supplementary file 5 — Figure S1: The species tree resulting from the ASTRAL‐III analysis of the restricted ortholog only dataset (paralog 1to1 ortholog dataset, dataset 2) where all loci with putative paralog warnings were excluded. Local posterior probabilities of clades are given (values in red indicate branches for which a polytomy could not be rejected). Pie charts indicate relative frequencies of alternative topologies around each branch (blue = congruent with species tree; yellow = first alternative topology; red = second alternative topology). Clade names are discussed in the text. Figure S2: The species tree resulting from the ASTRAL‐Pro analysis of the paralog AstralPro dataset (dataset 3). Local posterior probabilities of clades are given. Pie charts indicate relative frequencies of alternative topologies around each branch (blue = congruent with species tree; yellow = first alternative topology; red = second alternative topology). Clade names are discussed in the text. Figure S3: The species tree resulting from the ASTRAL‐III analysis of the exon supercontig standard dataset (dataset 4) with exons + regions flanking the exons from a single sequence per accession (i.e., the standard output from HybPiper). Local posterior probabilities of clades are given (values in red indicate branches for which a polytomy could not be rejected). Pie charts indicate relative frequencies of alternative topologies around each branch (blue = congruent with species tree; yellow = first alternative topology; red = second alternative topology). Clade names are discussed in the text. Figure S4: The species tree resulting from the ASTRAL‐III analysis of the exon supercontig recombination‐free dataset (dataset 5) where loci with evidence for recombination were removed. Local posterior probabilities of clades are given (values in red indicate branches for which a polytomy could not be rejected). Pie charts indicate relative frequencies of alternative topologies around each branch (blue = congruent with [file ECE3-16-e73863-s001.docx]

Supplementary Figures S1-S5

**Figure S1**. The species tree resulting from the ASTRAL-III analysis of the restricted ortholog only dataset (*paralog 1to1 ortholog* dataset, dataset 2) where all loci with putative paralog warnings were excluded. Local posterior probabilities of clades are given (values in red indicate branches for which a polytomy could not be rejected). Pie charts indicate relative frequencies of alternative topologies around each branch (blue = congruent with species tree; yellow = first alternative topology; red = second alternative topology). Clade names are discussed in the text.

**Figure S2**. The species tree resulting from the ASTRAL-Pro analysis of the *paralog AstralPro* dataset (dataset 3). Local posterior probabilities of clades are given. Pie charts indicate relative frequencies of alternative topologies around each branch (blue = congruent with species tree; yellow = first alternative topology; red = second alternative topology). Clade names are discussed in the text.

**Figure S3**. The species tree resulting from the ASTRAL-III analysis of the *exon supercontig standard* dataset (dataset 4) with exons + regions flanking the exons from a single sequence per accession (i.e., the standard output from HybPiper). Local posterior probabilities of clades are given (values in red indicate branches for which a polytomy could not be rejected). Pie charts indicate relative frequencies of alternative topologies around each branch (blue = congruent with species tree; yellow = first alternative topology; red = second alternative topology). Clade names are discussed in the text.

**Figure S4**. The species tree resulting from the ASTRAL-III analysis of the *exon supercontig recombination-free* dataset (dataset 5) where loci with evidence for recombination were removed. Local posterior probabilities of clades are given (values in red indicate branches for which a polytomy could not be rejected). Pie charts indicate relative frequencies of alternative topologies around each branch (blue = congruent with species tree; yellow = first alternative topology; red = second alternative topology). Clade names are discussed in the text.

**Figure S5**. The species tree resulting from the ASTRAL-III analysis of the *gene supercontig* dataset (dataset 6) with exons, introns + regions flanking the gene. Local posterior probabilities of clades are given (values in red indicate branches for which a polytomy could not be rejected). Pie charts indicate relative frequencies of alternative topologies around each branch (blue = congruent with species tree; yellow = first alternative topology; red = second alternative topology). Clade names are discussed in the text.
